# Supplementary material for: Content-rich biological network constructed by mining PubMed abstracts
Source: BMC Bioinformatics. 2004 Oct 8;5:147. doi: 10.1186/1471-2105-5-147 (PMC528731; doi:10.1186/1471-2105-5-147)
Supplement: Additional File 2 — The original results of the above study (non-essential files are deleted to keep the file size under the limit set by BMC bioinformatics). [file 1471-2105-5-147-S2.bz2 › chilibotAdditionalFile2/dip05/253ID7809159E1011/html/CKS1_CDK2.html]

 


 **CKS1** and **CDK2** 
  
Found 11 abstracts in PubMed, retrieved 05.  
 

 What does Google say? 
 PDF only 
| .edu only 

---

**Interactive relationship** (e.g. stimulation, inhibition, etc)

**Neutral relationship**- The interaction of Skp2 with the substrate is further strengthened by the association of the Cdk binding site of  **Cks1**  with  **Cdk2**  cyclin E, to which phosphorylated p27 is bound.  Ref: 12140288 J Biol Chem, 2002
- iii in vitro, the phosphorylated forms of CDK1 and  **CDK2**  were preferentially linked to CKS  [ **CKS1** ] .  Ref: 10360827 Int J Cancer, 1999

**Non-interactive relationship** (e.g. studied together, co-existance, homology, etc.)

- Despite the high degree of homology between the nine known CDKs, only CDK1,  **CDK2**  and, to a lesser extent, CDK3 are able to bind to the suc1 Cks  [ **CKS1** ]  proteins.  Ref: 12007796 Biochim Biophys Acta, 2002
- The ubiquitylation of p21 in vitro required the supplementation of all components of the SCF complex, as well as of  **Cks1**  and  **Cdk2**  cyclin E.  Ref: 12730199 J Biol Chem< MedlineTA>J Biol Chem, 2002
